# Preperimetric Glaucoma Prospective Observational Study (PPGPS): Design, baseline characteristics, and therapeutic effect of tafluprost in preperimetric glaucoma eye

**DOI:** 10.1371/journal.pone.0188692

**Published:** 2017-12-13

**Authors:** Naoko Aizawa, Hiroshi Kunikata, Yukihiro Shiga, Satoru Tsuda, Yu Yokoyama, Kazuko Omodaka, Tomoki Yasui, Keiichi Kato, Hiroaki Kurashima, Etsuyo Miyamoto, Masayo Hashimoto, Toru Nakazawa

**Affiliations:** 1 Department of Ophthalmology, Tohoku University Graduate School of Medicine, Sendai, Japan; 2 Department of Retinal Disease Control, Tohoku University Graduate School of Medicine, Sendai, Japan; 3 Department of Ophthalmic Imaging and Information Analytics, Tohoku University Graduate School of Medicine, Sendai, Japan; 4 Yasui Eye Clinic, Rifu, Japan; 5 Kato Eye Center, Taiwa, Japan; 6 Japan Medical Affairs, Global R&D, Santen Pharmaceutical Co. Ltd., Osaka, Japan; 7 Department of Advanced Ophthalmic Medicine, Tohoku University Graduate School of Medicine, Sendai, Japan; Universita degli Studi di Firenze, ITALY

## Abstract

**Purpose:**

There is no consensus on the diagnosis or treatment policy for Preperimetric Glaucoma (PPG) because the pathogenesis of PPG is not clear at this time. Preperimetric Glaucoma Prospective Observational Study (PPGPS) is a first multicenter, prospective, observational study to clarify the pathogenesis of PPG. This article indicates study design, patient baseline characteristics, and analysis focused on optic nerve head (ONH) blood flow in PPG, as well as the intraocular pressure (IOP) -lowering effect and ONH blood flow-improving effects of Tafluprost.

**Method:**

In this study, 122 eyes from 122 subjects (mean age: 53.1 ± 14.3) newly diagnosed as PPG were enrolled. The circumpapillary retinal nerve fiber layer thickness (cpRNFLT) was evaluated with optical coherence tomography (OCT). The ONH blood flow was measured with laser speckle flowgraphy. The therapeutic effect of Tafluprost was evaluated at Month 0 (ONH blood flow-improving effect) and Month 4 (IOP-lowering effect).

**Results:**

The untreated IOP, cpRNFLT, and baseline Mean deviation (MD) value was 16.4 ± 2.5 mmHg, 80.4 ± 8.2 μm, and -0.48 ± 1.29 dB, respectively. In the site-specific visual field evaluation using the sector map, there was no appreciable site-specific visual field defect in the eye with PPG. The inferior region of cpRNFLT in 4-quadrant OCT sector analysis and 6 o’clock region in 12-o’clock OCT sector analysis was the highest rate of abnormality in PPG eyes. Topical administration of Tafluprost significantly reduced IOP from 16.4 ± 2.5 mmHg at baseline to 14.5 ± 2.3 mmHg at Month 4 (*P* < 0.001, paired *t-test*). In the linear regression analysis, there was a significant relationship between the increase of ONH blood flow and baseline value.

**Conclusion:**

PPGPS is a first prospective study focusing on the pathology of PPG. This study is expected to elucidate the pathology of PPG, with evidence useful for determining a treatment strategy for PPG.

## Introduction

Glaucoma is a leading cause of visual impairment, with an estimated 60.5 million patients globally [1]. There is concern that the number of glaucoma patients will continue to increase with the worldwide population growth and progress of the aging society. According to an estimation based on demographic analysis, there may be 1.74 times more glaucoma patients in 2040 than in 2013 [2]. Considering the current situation that many glaucoma patients also have visual impairment, an increase in patients suffering from visual impairment is also likely to increase similarly in the future. Under these circumstances, social demand requires provision of early diagnosis and optimal treatment to individual patients.

Glaucoma is an irreversible, chronic, progressive optic neuropathy, which is characterized by visual field impairment that occurs secondarily to glaucomatous morphological changes in the optic nerve head (ONH) and the retinal nerve fiber layer (RNFL). Weinreb RN et al. proposed a concept named “Glaucoma Continuum,” indicating the progression process of glaucoma [3–4]. In this concept, the pathology of glaucoma can be presented as a series of “continuous events,” beginning with death of retinal ganglion cells (RGCs), then progressing to preperimetric glaucoma (PPG) with detectable morphological changes in ONH and RNFL, and leading to visual field impairment. A recent study by Medeiros FA et al. suggested that a substantial loss of RGCs occurs at the PPG stage [5]. Since glaucomatous morphological changes and visual field disturbances are irreversible, examination should be regularly performed to identify disease progression as early as possible during the PPG stage (before the disease progresses to visual field impairment) and thereby correctly evaluate whether treatment is necessary in consideration of risk factors.

Classically, glaucoma is diagnosed based on glaucomatous morphological changes (detected by funduscopy) or visual field impairment (measured using automated static perimetry). With the recent developments in examination technology, optical coherence tomography (OCT)-based fundus imaging and new perimetric techniques are available in daily practice. These technological innovations have allowed sensitive detection of glaucomatous morphological changes and visual field impairment even during the PPG stage, thereby enabling assessment of disease progression [6–9]. With regard to impairment of blood flow in the ONH (hereinafter, ONH blood flow impairment), of which the involvement in glaucoma progression has been suggested, the innovations have enabled non-invasive measurement of ocular blood flow parameters using laser speckle flowgraphy (LSFG) while ensuring high reproducibility [10–12]. These technological advancements are expected to contribute to elucidation of the pathogenesis of glaucoma. However, the pathogenesis of PPG is not clear at the present time, and thus there is no consensus on diagnosis or treatment policy for PPG.

We are conducting a multicenter, prospective, observational study in patients with PPG on a new treatment with a topical prostaglandin F receptor agonist. The purpose of this study is to elucidate the pathogenesis of PPG. This study was designed to prospectively assess disease progression in PPG using OCT-based morphological indices, as well as visual field impairment. With regard to therapeutic effects of the prostaglandin F receptor agonist, we intend to evaluate not only the change of intraocular pressure (IOP), but also ONH blood flow using LSFG. Here, we report the study design, patient baseline characteristics, and analysis focused on ONH blood flow in PPG, as well as IOP-lowering effect and ONH blood flow-improving effects of the topical prostaglandin F receptor agonist.

## Materials and methods

This was a multicenter, prospective, observational study. Subjects were longitudinally evaluated in accordance with a protocol that included regular follow-up visits (4-month intervals) for clinical examination and several other imaging and functional tests in part of routine clinical care. This research followed the tenets of the Declaration of Helsinki and was approved the Institutional Review Board of Tohoku Graduate School of Medicine (Protocol number: 2011–498). Documenting informed consent was obtained from all subjects, and written informed consent form, and written information was approved by the Institutional Review Board of Tohoku Graduate School of Medicine. Subject needed special consideration (minor, adult with impaired decision-making ability, adult in an unconscious state, adult requiring considerations for the name of his/her disease) was not enrolled in this study. This study is registered at UMIN Clinical Trials Registry with the identifier UMIN000013733.

### Subjects

Subjects were enrolled from Tohoku University Hospital, Kato Eye Center, and Yasui Eye Clinic from outpatient glaucoma services. Some of patients were previously treated with IOP-lowering agent, and these patients underwent wash-out (discontinue use of all IOP-lowering medications) over 1 month before baseline. Eligibility criteria were as follows: (1) age over 20 years or over; (2) open angle on gonioscopy (grade 3 or 4 in Shaffer classification); (3) refractive error within +3.00 to -8.00 diopters; (4) best-corrected visual acuity better than 20/20; (5) IOP 21 mmHg or less in at least three examinations; (6) abnormal circumpapillary RNFL thickness (cpRNFLT) in at least one clockwise OCT scan sector between 6, 7, 8, 10, 11, and 12 o’clock (6, 5, 4, 2, 1 and 0 o’clock in left eye), confirmed in at least there examinations; (7) visual field within the normal limits of glaucoma hemifield test with pattern standard deviation (PSD) greater than 5%, confirmed in at least two examinations. Exclusion criteria were the following: (8) having corneal abnormalities or other conditions preventing reliable applanation tonometry; (9) having retinal diseases affecting the retinal nerve fiber layer thickness; (10) having the finding of secondary glaucoma. In case both eyes of a subject met all inclusion criteria, the eye with thinner cpRNFLT was enrolled in this study. All subjects enrolled in this study were considered applicable for starting on glaucoma medication by physicians and were topically treated with prostaglandin F receptor agonist.

### Measurement of clinical parameters

Subjects underwent an ophthalmological and general examination, which comprised the following: slit lamp and funduscopic examination, gonioscopy, IOP measurement, systemic blood pressure measurement, visual field examination, OCT examination, and ONH blood flow assessment. IOP was determined with Goldmann applanation tonometry under local anesthesia. The systolic blood pressure (SBP) and diastolic blood pressure (DBP) were measured according to the standard technique, in the brachial artery at the height of the heart with an automated monitor. Mean arterial blood pressure (MBP) and ocular perfusion (OPP) were calculated as follows: MBP = DBP + 1/3 (SBP–DBP), and OPP = 2/3 MBP—IOP.

#### Visual field evaluation and OCT examination

**Fig 1:**
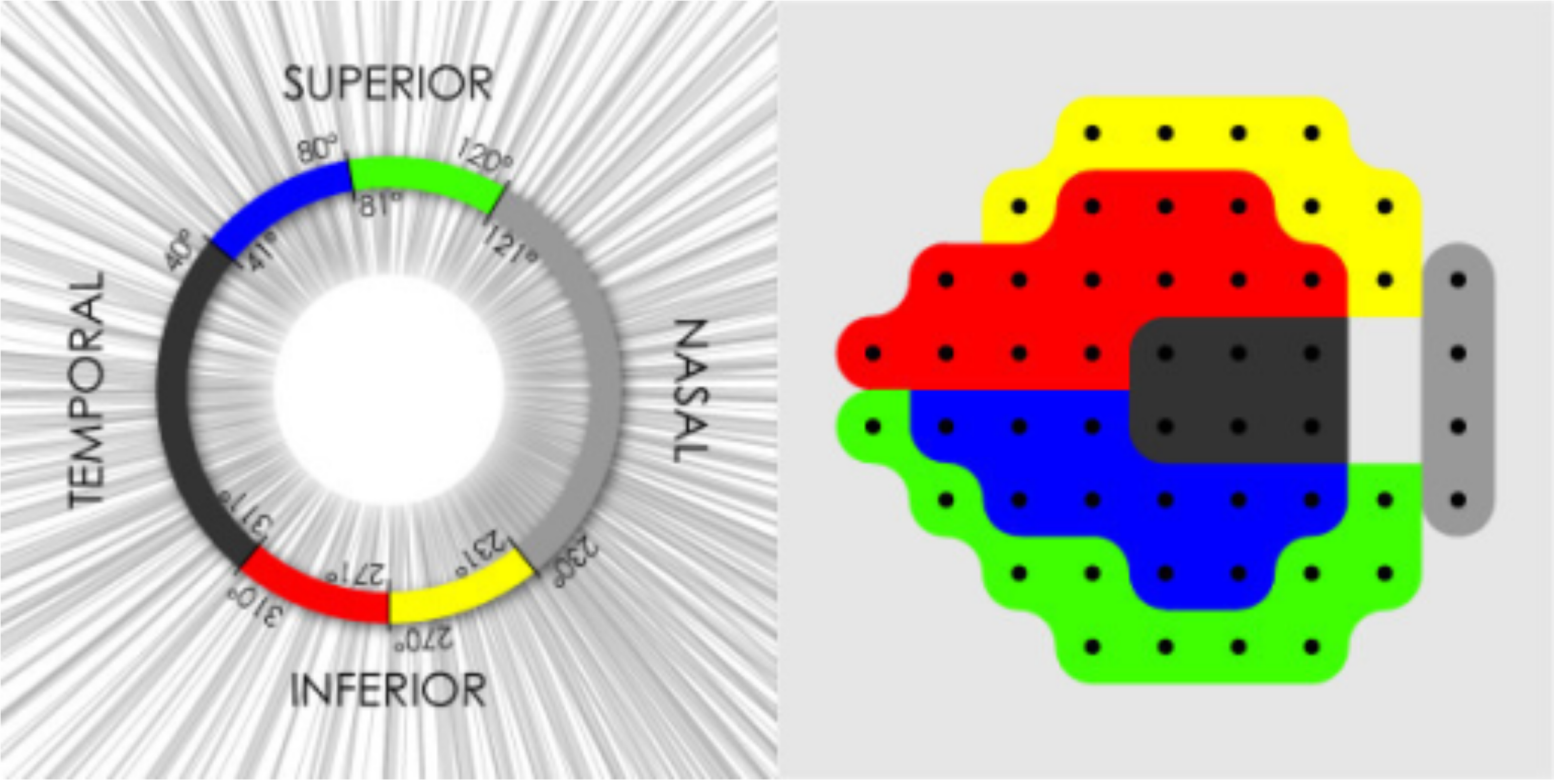
Map representing the relationship between Standard Automated Perimetry visual field sectors and sections of the peripapillary OCT scan circle. Bizies D et al. Integration and fusion of standard automated perimetry and optical coherence tomography data for improved automated glaucoma diagnostics. BMC Ophthalmol 2011; 11: 20 2011 Bizies et al; licensee Bio Med Central Ltd. Creative Commons Attribution License (https://creativecommons.org/licenses/by/2.0/).

Visual field was measured by static automated perimetry using the Swedish Interactive Threshold Algorithm (Standard 24–2) of a Humphrey Field Analyzer (Carl Zeiss Meditec Inc., Dublin, California). Visual field measurements with fixation losses of more than 20%, or with over 33% false positives or false negatives were excluded from the analysis. Mean deviation (MD) and total deviation (TD) were used for the evaluation of visual field function. The site-specific visual field progression was evaluated using the visual field sector map established by Garway-Heath DF [13], relating visual field test points to regions of the ONH (Fig 1). Spectral-domain OCT (Cirrus HD-OCT, Carl Zeiss Meditec Inc., Dublin, California) was used for the evaluation of cpRNFLT. Images with signal strength less than 6 were considered of poor quality and excluded from the data analysis. All OCT data were masked and reviewed at a reading center organized by experienced physicians of Tohoku University Graduate School of Medicine to evaluate scan error such as segmentation error, blink artifacts, and out-of-register artifacts. The cpRNFLT, the RNFLT in 4 quadrants, and 12-o’clock positions were used in the evaluation. RNFL defects were assessed clockwise in the right eyes and counterclockwise in the left eyes. In the 12 sector analysis at ONH, 7, 8, 10, 11, and 12 o’clock sectors in right eye is correspondence to 5, 4, 2, 1 and 0 o’clock sectors in left eye, respectively. The OCT software automatically classified all RFNLT as within normal limits or abnormal (out of 95 percentile from age-matched healthy eye).

#### ONH blood flow assessment with LSFG

The ocular blood flow at ONH was measured using LSFG-NAVI (Softcare Co., Ltd., Fukutsu, Japan), which has been approved by the Pharmaceuticals and Medical Devices Agency in Japan. The detailed principles of LSFG have been described in previous reports [10]. Mean blur rate (MBR), an index of relative blood flow velocity, was used for the evaluation of ocular blood circulation. The LSFG software automatically divides the large vessel and capillary tissue areas. In this study, MBR in capillary tissue areas (MBR_T_) was used, since its usefulness in intergroup comparison has been reported [11–12]. The pupils of each subject were dilated with 0.4% tropicamide (Mydrin M^®^; Santen Pharmaceutical Co. Ltd, Osaka, Japan) before LSFG measurement. The measurement was conducted three times at each time point, and the average MBRs were used for analyses. Data indicating poor image quality were excluded from the analysis. Blinded assessments of all images were conducted at a reading center organized by experienced technicians of Softcare Co., Ltd. and physicians of Tohoku University.

### Evaluation of therapeutic effect of prostaglandin F receptor agonist on ONH blood flow and IOP in PPG eyes

The therapeutic effect of prostaglandin F receptor agonist was evaluated at Month 0 and Month 4. The changes of IOP, ONH blood flow, and OPP were measured before administration and 90–120 minutes after administration of topical prostaglandin F receptor agonist at Month 0. Tafluprost ophthalmic solution 0.0015% (TAPROS^®^; Santen Pharmaceutical, Osaka, Japan) is a selective prostaglandin F receptor agonist, and topical administration of tafluprost reduces IOP in glaucoma patients. In addition, tafluprost improved the ONH blood flow in glaucoma patients, as we previously reported [14]. Therefore, the therapeutic effect of tafluprost ophthalmic solution 0.0015% was evaluated in this analysis. The therapeutic effect on IOP and OPP was evaluated at Month 4. All enrolled subjects were treated once daily with tafluprost ophthalmic solution 0.0015% for 4 months.

### Statistical analysis

The data are described as mean ± standard deviation or number (percentage). Paired *t*-test was used for evaluation of therapeutic effect from baseline. Linear regression analysis and multiple linear regression analysis were conducted in this study. In linear regression analysis, the test for Pearson’s correlation coefficient was performed. Multiple linear regression analysis was applied to determine variables affecting the change of visual field progression. Statistical analysis was performed using SAS version 9.4 (SAS Institute Inc., Cary, NC, USA). The significance level was set at *P* < 0.05.

## Results

### Demographic and ocular characteristics in PPG eyes

**Fig 2:**
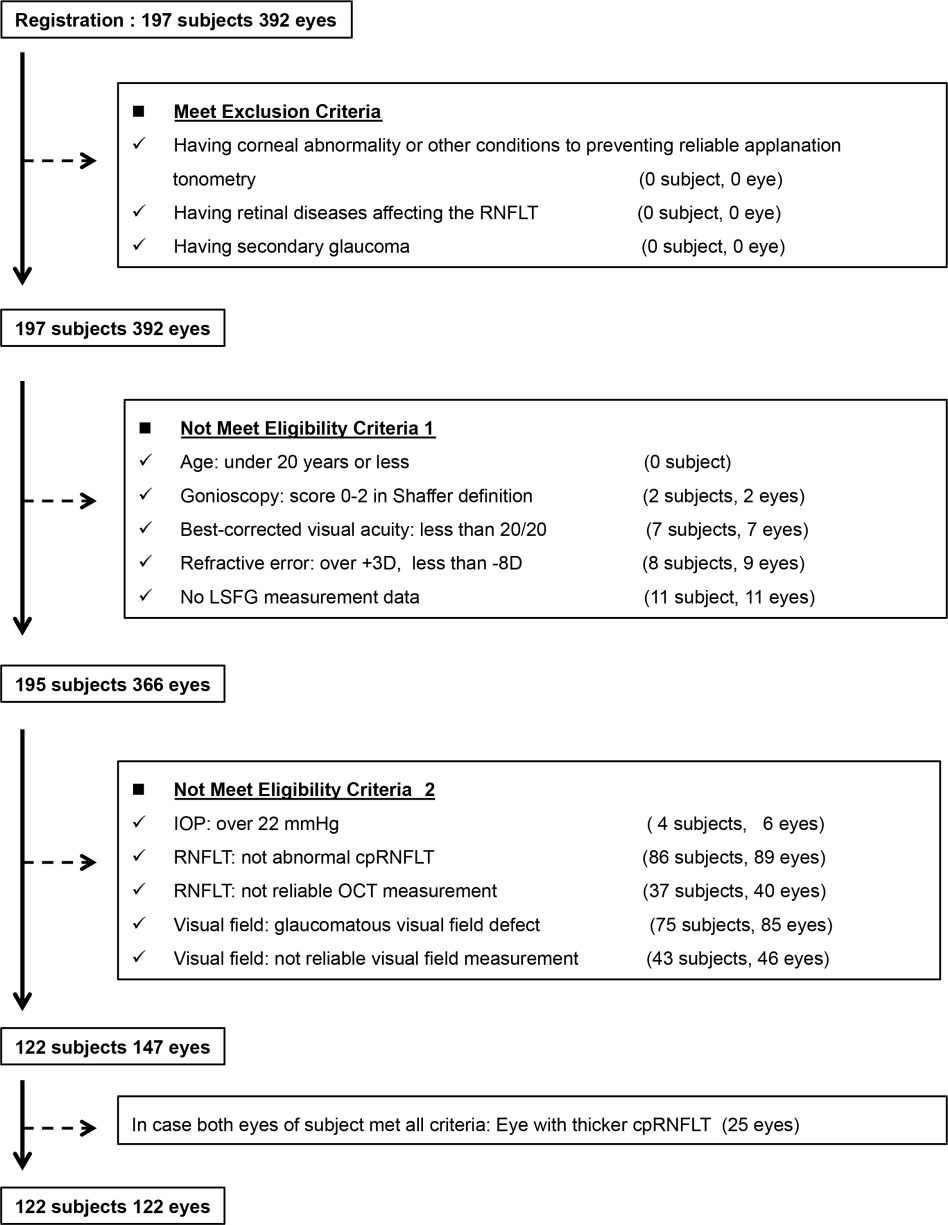
Participant flow diagram.

In this study, 122 eyes from 122 subjects beginning mono-therapy with tafluprost ophthalmic solution 0.0015% were identified between August 2012 and December 2014 (Fig 2). Table 1 indicates the baseline demographic and ocular characteristic (age, sex, untreated IOP, Spherical equivalent, MBP, OPP, cpRNFLT), complications (hypertension, hyperlipidemia, diabetes, feeling of cold and migraine), and visual field parameters. The untreated IOP, cpRNFLT, and baseline MD value was 16.4 ± 2.5 mmHg, 80.4 ± 8.2 μm, and -0.48 ± 1.29 dB, respectively. In the site-specific visual field evaluation using the sector map defined by Garway-Heath DF, there was no appreciable site-specific visual field defect in the eye with PPG.

**Table 1:** Baseline demographic/characteristics of preperimetric glaucoma eye.

	Number of eyes		n = 122
Demographic characteristics	Age	(year)	53.1 ± 14.3
	Female	n (%)	54 (44.3%)
	Untreated IOP	(mmHg)	16.4 ± 2.5
	Spherical equivalent	(diopter)	-2.13 ± 2.78
	MBP	(mmHg)	89.0 ± 15.2
	OPP	(mmHg)	43.0 ± 10.2
	cpRNFLT	(μm)	80.4 ± 8.2
Complications	Hypertension	n (%)	33 (27.0%)
	Hyperlipidemia	n (%)	21 (17.2%)
	Diabetes	n (%)	9 (7.4%)
	Feeling of cold	n (%)	19 (15.6%)
	Migraine	n (%)	13 (10.7%)
Visual field parameters	MD	(dB)	-0.48 ± 1.29
	TD	(dB)	-2.58 ± 8.12
	TD-_Central_	(dB)	-0.28 ± 1.42
	TD- _Inferior/Temporal_	(dB)	-0.74 ± 1.31
	TD-_Inferior/Nasal_	(dB)	-0.56 ± 1.43
	TD-_Nasal_	(dB)	-0.34 ± 1.82
	TD-_Superior/Nasal_	(dB)	-0.18 ± 1.72
	TD-_Superior/Temporal_	(dB)	-0.48 ± 1.45
	TD-Inferior	(dB)	-1.31 ± 2.63
	TD-Superior	(dB)	-0.66 ± 3.01

Values are means ± standard deviations. AU = arbitrary unit, IOP = intraocular pressure, MBP = mean blood pressure, OPP = ocular perfusion pressure, cpRNFLT = circumpapillary retinal nerve fiber layer thickness, MD = mean deviation, TD = total deviation, ONH = optic nerve head, MBRT = Mean blur rate at tissue

The average value and percentage of abnormal eyes (out of 95 percentile from age-matched healthy eye) for cpRNFLT in each “4-quadrant sector analysis” and “12-o’clock sector analysis” are shown in Table 2. The inferior region in 4-quadrant sector analysis and 6 o’clock region in 12-o’clock sector analysis was the highest rate of abnormality in PPG eyes. The percentage of abnormal eye at inferior region in 4-quadrant sector analysis and at 6 o’clock in 12-o’clock sector analysis was 43.4% and 45.9%, respectively.

**Table 2:** Abnormality of circumpapillary retinal nerve fiber layer thickness at baseline in preperimetric glaucoma eyes.

		Average thickness (μm)	Abnormal eyes (outside 95% percentile)
4 quadrants	Superior	96.9 ± 16.5	n = 53 (43.4%)
	Temporal	63.2 ± 13.4	n = 14 (11.5%)
	Inferior	99.7 ± 15.0	n = 55 (45.1%)
	Nasal	61.5 ± 9.6	n = 17 (13.9%)
12 clock-hour sectors	6 o’clock	101.3 ± 25.9	n = 56 (45.9%)
	7 o’clock	117.8 ± 24.2	n = 21 (17.2%)
	8 o’clock	68.0 ± 17.5	n = 7 (5.7%)
	9 o’clock	50.0 ± 10.0	n = 13 (10.7%)
	10 o’clock	71.7 ± 18.7	n = 24 (19.7%)
	11 o’clock	107.0 ± 25.9	n = 40 (32.8%)
	12 o’clock	94.8 ± 25.3	n = 41 (33.6%)

Values are means ± standard deviations. The directional angle was evaluated in a clockwise direction in right eyes and in a counterclockwise direction in left eyes, with the temporal equator configures at 0°. In the 12 sector analysis at ONH, 7, 8, 10, 11, and 12 o’clock sectors in right eye is correspondence to 5, 4, 2, 1 and 0 o’clock sectors in left eye, respectively.

### Characteristics of ONH blood flow in PPG eyes

The MBR_T_ was 11.50 ± 2.14 overall, 11.85 ± 2.48 at superior region, 9.36 ± 2.29 at temporal region, 12.67 ± 2.47 at inferior region, and 14.02 ± 2.58 AU at nasal region, respectively. Table 3 indicates the relationship between overall MBR_T_ and patient background in linear regression analysis. The significant relationships between overall MBR_T_ and OPP (r = -0.196, *P* = 0.030) and between overall MBR_T_ and cpRNFLT (r = 0.248, *P* = 0.006) were noted. On the other hand, there was no significant relationship between overall MBR_T_ and age, untreated IOP, or spherical equivalent.

**Table 3:** Relationship between ocular blood flow and patient background parameter at baseline in preperimetric glaucoma eyes.

		r	*P* value
MBR_T_ (AU)	Age (year)	-0.153	0.092
	Untreated IOP (mmHg)	-0.133	0.144
	Spherical equivalent (diopter)	-0.163	0.072
	OPP (mmHg)	-0.196	0.030
	cpRNFLT (μm)	0.248	0.006

IOP = Intraocular pressure, OPP = ocular perfusion pressure, MBRT = mean blur rate at tissue, AU = arbitrary unit, cpRNFLT = circumpapillary retinal nerve fiber layer thickness

### Effect of Tafluprost topical administration on IOP, OPP, and ONH blood flow in PPG eyes

All analyzed patients were topically treated with tafluprost ophthalmic solution 0.0015% once daily, and IOP and blood pressure was evaluated at Month 4. Topical administration of tafluprost significantly reduced IOP from 16.4 ± 2.5 mmHg at baseline to 14.5 ± 2.3 mmHg at Month 4 (*P* < 0.001, paired *t*-test). In addition, OPP significantly increased from 43.0 ± 10.2 mmHg at baseline to 45.3 ± 9.3 mmHg at Month 4 (*P* = 0.001).

**Fig 3:**
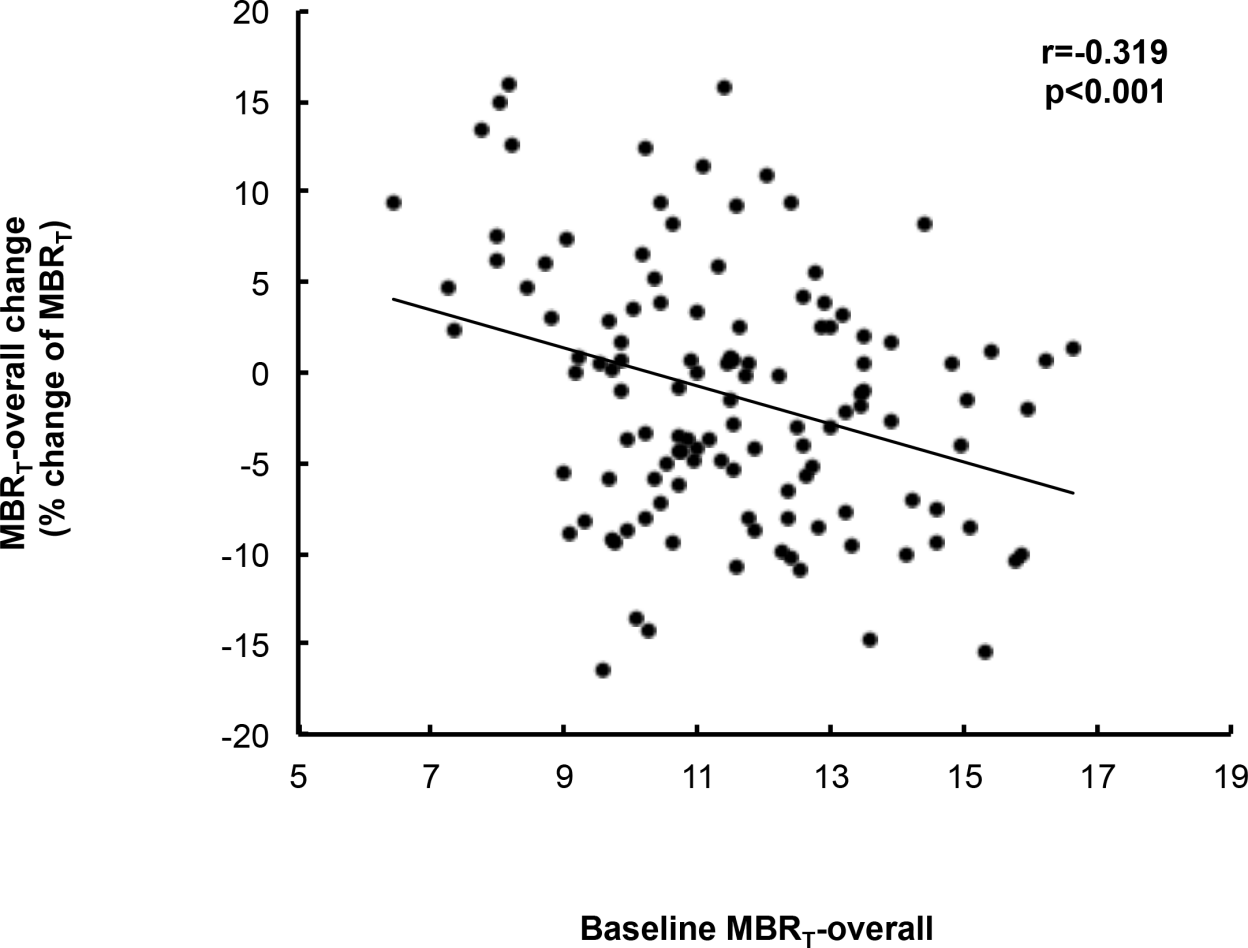
Relationship between baseline ocular blood flow and change of ocular blood flow after topical prostaglandin analogue administration in preperimetric glaucoma eyes.

In addition, therapeutic effect of tafluprost ophthalmic solution on ONH blood flow was evaluated at Month 0. The percent change of overall MBR_T_ was -1.28 ± 7.04%. Fig 3 indicates a scatter plot demonstrating percent change of overall MBR_T_ on baseline overall MBR_T_. In the linear regression analysis, there was a significant relationship between percent change of overall MBR_T_ and baseline overall MBR_T_ (r = -0.319, *P* < 0.001). The involvement of demographic and ocular characteristic parameters in overall MBR_T_ was evaluated using multiple linear regression analysis. Before multiple linear regression analysis, multicollinearity among the variables was confirmed. There was strong correlation between age and spherical equivalent (r = 0.544, *P* < 0.001) and between MBP and OPP (r = 0.971, *P* < 0.001). Therefore, spherical equivalent and MBP were excluded from the candidate variables in multiple linear regression analysis. Table 4 shows the results of multiple linear regression analysis, in which the independent variables were age, sex (female), IOP, OPP, cpRNFLT, and MBR_T_-overall. MBR_T_-overall was detected as the only significant effective factor in percent change of MBR_T_-overall (β = -0.317, 95% confidence interval -1.652–0.438, *P* < 0.001). On the other hand, the influence of age, sex (female), IOP, OPP, and cpRNFLT-Inferior were not determined as significant potent parameters for percent change of MBR_T_-overall. The correlation analysis and multiple linear regression analysis using log-transformed MBR-t indicated the similar to that using non-log-transformed MBR-t (S1 Fig and S1 Table).

**Table 4:** Multiple linear regression analysis for change of ocular blood flow 2 hours after topical prostaglandin analogue administration in preperimetric glaucoma eyes.

Variable				
Dependent	Independent	β	(95% Cl)	*P* value
ΔMBR_T_ (AU)	Age (10 years)	0.011	(-0.836, 0.940)	0.908
	Female	-0.066	(-3.488, 1.612)	0.468
	IOP (3 mmHg)	-0.005	(-1.578, 1.500)	0.960
	OPP (5 mmHg)	-0.125	(-1.076, 0.212)	0.186
	cpRNFLT (5 μm)	-0.069	(-1.093, 0.494)	0.456
	MBR_T_−Overall (1 AU)	-0.317	(-1.652, -0.438)	<0.001

IOP = Intraocular pressure, OPP = ocular perfusion pressure, MBRT = mean blur rate at tissue, AU = arbitrary unit, cpRNFLT = circumpapillary retinal nerve fiber layer thickness

## Discussion

There is no consensus on diagnosis or treatment policy for PPG because the pathogenesis of PPG is not clear at this time. Although several studies focused on the pathology of PPG have been previously reported, these were cross-sectional or retrospective studies [15–16]. The cross-sectional study provides a 'snapshot' of the cause and outcome. Therefore, it is not inadequacy to discuss true cause and outcome relationship without longitudinal study. In addition, retrospective study may be susceptible to selection bias and/or information bias, and thus caution should be taken when interpreting the study results. Under these circumstances, we have decided to implement a first prospective study focusing on PPG pathology. Here we report the study design, patient baseline characteristics, and analysis focused on parameters related to ONH blood flow in PPG, as well as evaluate the IOP-lowering and ONH blood flow-improving effects of tafluprost. In the analysis focused on ONH blood flow parameters, a strong correlation between the ONH blood flow and cpRNFLT was demonstrated in PPG eyes. In addition, tafluprost shows significant IOP-lowering effect as well as an ONH blood flow-improving effect in eyes with ONH blood flow impairment.

PPG is defined as no glaucomatous visual field defect, and the various reports have been published on criteria for diagnosing glaucomatous visual field impairment [17–19]. In the present study, eyes within normal limitation in glaucoma hemifield test and with a PSD greater than 5% were included as those without glaucomatous visual field impairment. The visual field impairment often appears locally in early-stage glaucoma, and it is important to sensitively detect local visual field defect. Therefore, we evaluated the visual field function based not only on global visual field indices (MD value), but also on site-specific visual field sector analysis established by Garway-Heath DF et al. [13]. The baseline MD was −0.48 ± 1.29 dB in all the eyes enrolled in the study. Furthermore, there was no visual field impairment in visual field sector analysis. These findings suggested that the eyes enrolled in the study were in PPG stage.

There have been various reports on criteria for glaucomatous morphological changes in the ONH and RNFL; however, there is still no clear consensus at the present time. The discrimination ability of OCT parameter was evaluated in a previous report [20]. In this report, 12 sector analysis at ONH, particularly analyses of 6, 7, 8, 10, 11, and 12 o’clock sectors (in a counterclockwise direction in left eyes, with the temporal equator configures at 0°: 6, 5, 4, 2, 1 and 0 o’clock sectors in left eye), was suggested to provide useful parameters in terms of the accuracy of discriminating PPG eyes from healthy eyes. The present study was therefore designed to determine the presence or absence of glaucomatous morphological changes basically in these sectors. Consequently, thinning of cpRNFLT in the 6 o’clock sector was observed most frequently in the eyes enrolled in the present study, with a prevalence of 45.9%. In the above-mentioned discrimination analysis, the 6 o’clock to 7 o’clock sectors (temporal-inferior region, 6 o’clock to 5 o’clock in the left eye) provided the highest accuracy of discriminating PPG eyes. These results suggest that, temporal-inferior region at ONH was considered likely to susceptible to glaucomatous morphological changes during the PPG stage.

Various previous studies have suggested the involvement of ONH blood flow impairment in the pathology of glaucoma [21–22]. However, these previous studies have also revealed problems, such as poor measurement reproducibility, with the devices that have been used for ONH blood flow measurements [23]. LSFG is useful equipment, which allows non-invasive and simple measurement of fundus blood flow velocity. In addition, we conducted a cross-sectional study focused on analysis of ONH blood flow in healthy eyes, PPG eyes, and early-stage glaucoma eyes using LSFG [24]. The observed overall MBR_T_ values were 13.5 ± 2.6, 11.8 ± 2.4, and 11.0 ± 2.0 AU, respectively, in the healthy, PPG, and early-stage glaucoma eyes. In the present study, the overall MBR_T_ was determined to be 11.50 ± 2.14 AU for the PPG eyes enrolled, which was comparable to the value reported previously. This finding suggested that ONH blood flow was impaired in the eye with PPG. In the present study, a single correlation analysis was performed to clarify the parameters associated with ONH blood flow impairment in PPG. The analysis showed a strong correlation, particularly between the overall MBR_T_ and cpRNFLT, which was consistent with the results from our previous cross-sectional study [24]. Based on these findings, the PPG eye with thinning of the cpRNFL should be carefully managed in daily practice, because ONH blood flow impairment may have already occurred in these eyes.

Glaucoma is a chronic and irreversible disease, and lifetime treatment is generally necessary in glaucoma patients. Therefore, glaucoma medications require not only efficacy, but also safety and convenience. The current guidelines recommend the use of a prostaglandin-related drug as a first-line therapy for glaucoma, because well-balanced efficacy, safety, and convenience can be expected with these drugs [25–27]. Accordingly, subjects who had started therapy with prostaglandin F receptor agonist were enrolled in the present study. Here we report the IOP-lowering and the ONH blood flow-improving effects of tafluprost. Considering that it would generally take several weeks to 1 month for the stable IOP-lowering effects of the prostaglandin F receptor agonist, the IOP-lowering effect was evaluated at Month 4 in this study [28]. Significant IOP-lowering effect was observed, along with a significant increase in OPP. The ONH blood flow-improving effect was evaluated at Month 0, because our previous report demonstrated that the effect of tafluprost on ONH blood flow was clearly detectable 90–120 minutes after a single administration [14]. Although the improvement of ONH blood flow was not observed overall, multiple linear regression analysis revealed a significant negative correlation between the change of overall MBR_T_ and the baseline overall MBR_T_. The observed correlation indicates that tafluprost has an ONH blood flow-improving effect in eyes with ONH blood flow impairment. In these previous reports, intra-class correlation coefficient (ICC) and coefficient of variation (COV) for MBR measured by LSFG-NAVI were 0.92 to 0.98 and < 4.6%, respectively [11]. It has been also reported that the ICC and COV for cpRNFLT measured by Stratus OCT were 0.79 to 0.98 and < 4.2%, respectively [29]. Based on these previous reports, we consider that the reproducibility of LSFG-NAVI is sufficient to evaluate the ocular blood flow.

Endothelin-1 (ET-1) is suggested to be involved in the impairment of ONH blood flow observed in glaucoma eyes. ET-1 is a small peptide derived from vascular endothelial cells, which stimulates the endothelin type-A (ET_A_) and type-B (ET_B_) receptors [30–31]. In vascular smooth muscle cells, ET_A_ receptor stimulation causes the activation of voltage-dependent calcium (Ca^2+^) channels and elevates intracellular Ca^2+^ concentrations, causing vascular constriction [32]. In the result of a meta-analysis reported recently, the ET-1 concentrations in plasma and aqueous humor were found to be higher in glaucoma patients than in healthy adult subjects [33]. In addition, intravitreal injection of ET-1 causes impairment of ONH blood flow, glaucomatous morphological change at ONH, and RGCs death in rabbit [34]. These findings suggest an implication of ET-1 in glaucomatous ONH blood flow impairment. Previous study suggested that the ONH blood flow-improving effect of tafluprost would occur through the inhibition of voltage-dependent Ca^2+^ channels activation [35]. In addition, another non-clinical evaluation revealed that tafluprost prevented ET-1-induced ONH blood flow impairment and retinal degeneration [36–37]. In the present study tafluprost shows ONH blood flow-improving effect in eyes with ONH blood flow impairment. Based on these findings, tafluprost was suggested as possibly providing a beneficial therapeutic effect in eyes with ONH blood flow impairment caused by ET-1.

There are several limitations in the present study. First, all the study subjects had already started treatment with the prostaglandin F receptor agonist, and neither a placebo nor a non-treatment control group was set in the study. It is therefore difficult to discuss the therapeutic effects through comparison with natural clinical courses of PPG without treatment, based solely on the present study results. Second, the present report was focused on analysis of the therapeutic effects of prostaglandin F receptor agonist; however, the analysis was based entirely on only 4 month follow-up outcome. It is therefore necessary to further long-term evaluation in currently planned prospective observational study, and thereby evaluate the therapeutic effects of the prostaglandin F receptor agonist. Third, the present report only indicated the therapeutic effect of tafluprost in PPG. The enrollment of the subject treated with other prostaglandin F receptor agonist (latanoprost and travoprost) is small relative to total enrolled subject in this study. Although the enrollment criteria in the present study is subject under treatment with prostaglandin F receptor agonist, all subject enrolled in the present analysis were treated with tafluprost. Previous reports suggested the difference of pharmacological profile among prostaglandin F receptor agonist [36]. It is therefore difficult to discuss the therapeutic effect of latanoprost or travoprost in PPG eyes, based solely on the present study results. Forth, as mentioned above, the anti-ET-1 effect of tafluprost is discussed in this article; however, there has been no clinical evidence focused on the involvement of ET-1 in the pathology of PPG at the present time. Therefore, studies focused on the distribution of ET-1 in PPG eyes should be implemented to develop the discussion described in this article. Fifth, this study is observational study, and it is ethically difficult to implement an intervention to enrolled subjects. This means that intervention for the drug administration timing (morning administration or evening administration) during study period and observational timing at every visit is difficult in the present observational study. Therefore we evaluated the response to drugs in individual subject at Month 0 (before and 90–120 minutes after administration). In the linear regression analysis, the correlativity between percent change of overall MBR_T_ and baseline overall MBR_T_ was observed at Month 4, though the correlativity was not statistically significant (r = -0.166, p = 0.078). It seems that this unclear data was delivered from the difference of dosing and observational timing in individual subject. Therefore we defined the response to drugs in individual subject using the data obtained from Month 0 in the present study. Sixth, this is a first prospective study focusing on PPG, and there is no reference study at this time. Therefore it is difficult to have clear statistical hypothesis for sample size estimation, and we referred previous prospective clinical trial. Low-pressure Glaucoma Treatment Study prospectively evaluated the involvement of risk factor for visual field progression in NTG patients under treatment with IOP-lowering agents, and 193 subjects were enrolled in this study. The involvement of risk factor for visual field progression was evaluated in 127 subjects, and the association between ocular perfusion pressure and visual field progression as IOP-independent factor was suggested in the study [38]. Based on this previous study, we consider that the sample size of the present study is reasonable scale to evaluate the pathology of PPG. Glaucoma is multifactorial disease, and confounding among the risk factor should be adequately controlled in the statistical analysis. Therefore we are planning to use adequate statistical approach such as multivariable analysis for trend-based approach and Cox proportional-hazards analysis for event-based approach. Lastly, there is no clear consensus for the diagnosis of PPG. In the present study, no glaucomatous visual field defect is defined as visual field within the normal limits of glaucoma hemifield test with PSD greater than 5%, but the adequacy of the criteria is notclear at this time. The discussion and establishment about the diagnosis criteria of PPG should be needed near future.

In this article, regarding the prospective observational study currently underway while focusing on PPG, we reported the study design, patient baseline characteristics, and analysis focused on parameters related to ONH blood flow, as well as the IOP-lowering and ONH blood flow-improving effects of tafluprost. The study is expected to elucidate the pathology of PPG, with evidence useful for determining a treatment strategy for PPG.

## Supporting information

S1 Fig Relationship between baseline ocular blood flow (Log-transformed MBRt-overall) and change of ocular blood flow after topical prostaglandin analogue administration in preperimetric glaucoma eyes. MBRt-overall = mean blur rate at tissue MBRt-overall is log-transformed in this analysis.
(TIF)

S2 Fig Certificate of proofreading. This manuscript was edited faithfully and accurately by third party.
(TIF)

S1 Table Multiple linear regression analysis for change of ocular blood flow 2 hours after topical prostaglandin analogue administration in preperimetric glaucoma eyes. IOP = Intraocular pressure, OPP = ocular perfusion pressure, MBRT = mean blur rate at tissue, AU = arbitrary unit, cpRNFLT = circumpapillary retinal nerve fiber layer thickness MBRt-overall is log-transformed in this analysis
(TIF)
