# Supplementary figures and images for: Preperimetric Glaucoma Prospective Observational Study (PPGPS): Design, baseline characteristics, and therapeutic effect of tafluprost in preperimetric glaucoma eye
Source: PLoS One. 2017 Dec 13;12(12):e0188692. doi: 10.1371/journal.pone.0188692 (PMC5728557; doi:10.1371/journal.pone.0188692)

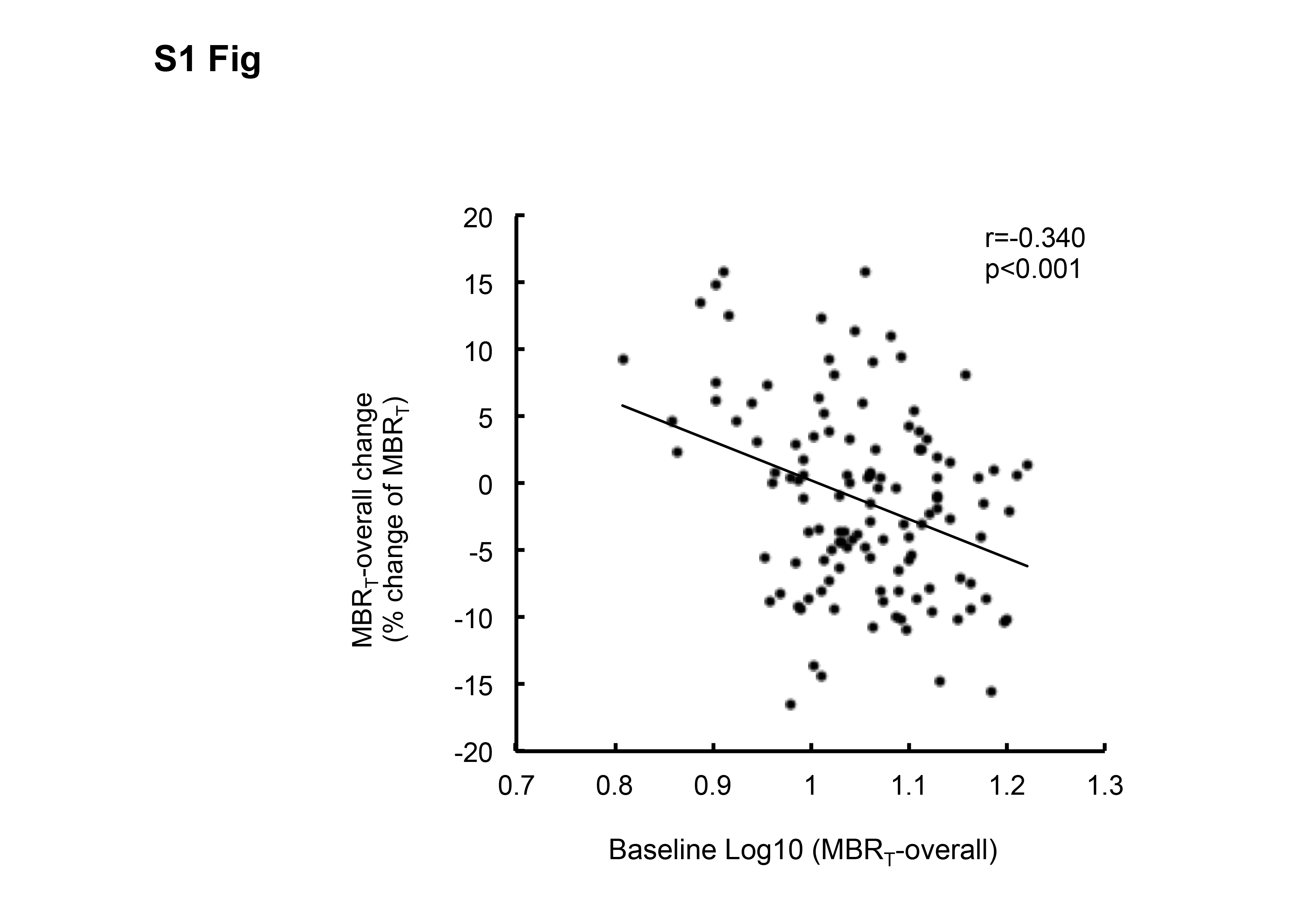

Supplement: S1 Fig — MBRt-overall = mean blur rate at tissue MBRt-overall is log-transformed in this analysis. (TIF) [file pone.0188692.s001.tif]

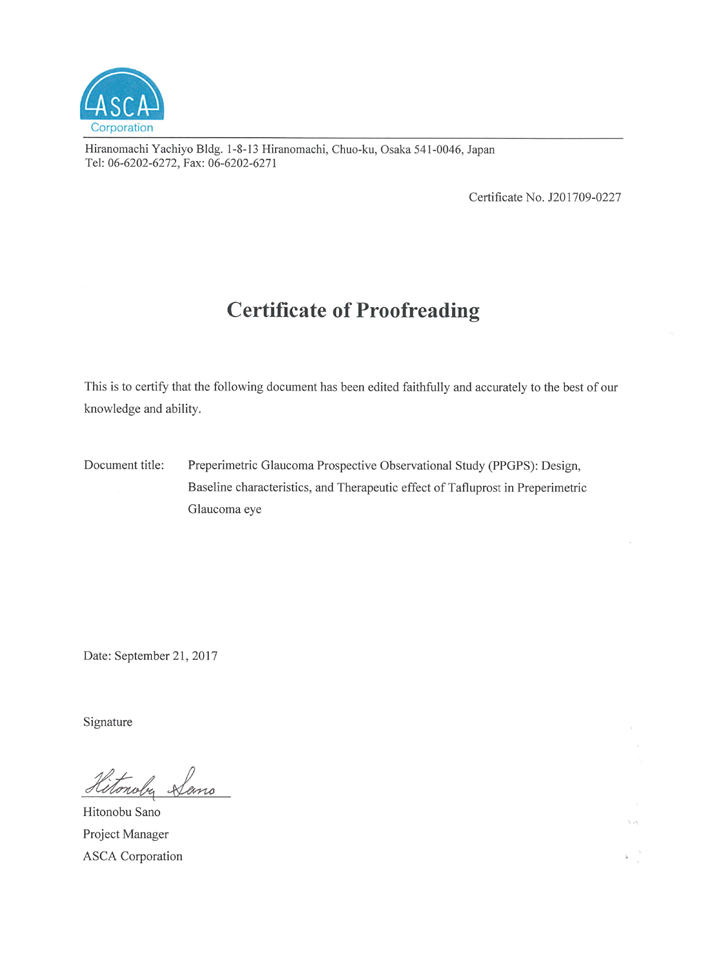

Supplement: S2 Fig — This manuscript was edited faithfully and accurately by third party. (TIF) [file pone.0188692.s002.tif]

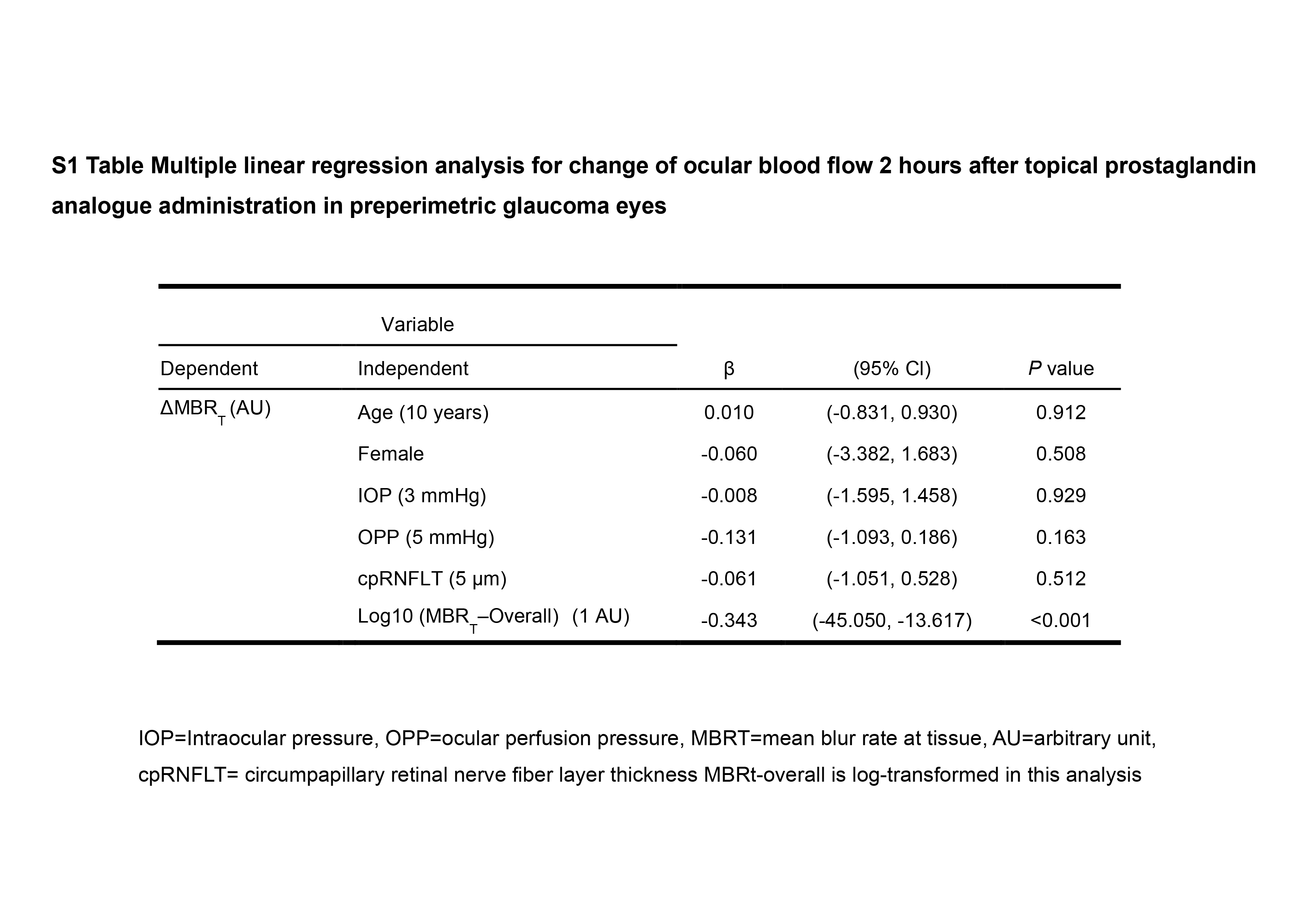

Supplement: S1 Table — IOP = Intraocular pressure, OPP = ocular perfusion pressure, MBRT = mean blur rate at tissue, AU = arbitrary unit, cpRNFLT = circumpapillary retinal nerve fiber layer thickness MBRt-overall is log-transformed in this analysis (TIF) [file pone.0188692.s003.tif]
